# Supplementary material for: Associations of Serum Folate and Holotranscobalamin with Cardiometabolic Risk Factors in Rural and Urban Cameroon
Source: Nutrients. 2021 Dec 30;14(1):178. doi: 10.3390/nu14010178 (PMC8747568; doi:10.3390/nu14010178)
Supplement: Supplementary file 1 [file nutrients-14-00178-s001.zip › nutrients-1488448-supplementary.pdf]

Supplementary material

Table S1: Socio-demographic characteristics of the study population stratified by sex and urban/rural residence, Cameroon study (n=578)

Characteristics	Women (n=370)			Men (n=208)		
	Rural (n=172)	Urban (n=198)	p-value	Rural (n=97)	Urban (n=111)	p-value
Age (years)	39.9± 8.1	38.6± 8.8	0.137	37.3±8.3	36.2±8.9	0.373
Education (years)	8.0±4.2	11.9±5.2	<0.001	8.9±4.5	13.3±5.2	<0.001
Education level, n (%) (completed)						
< Primary education	53(30.8)	24(12.1)		22(22.9)	4(3.6)	
Primary education	92(53.5)	74(37.4)	< 0.001	55(57.3)	32(28.8)	< 0.001
Secondary and high school	24(14.0)	66(33.3)		15(15.6)	41(36.9)	
University	3(1.7)	34(17.2)		4(4.2)	34(30.6)	
Alcohol intake, n (%)						
Never	26(15.1)	24(12.1)		10(10.3)	06(5.4)	
Past	17(9.9)	26(13.1)	0.485	03(3.1)	10(9.0)	0.104
Current	129(75.0)	148(74.8)		84(86.6)	95(85.6)	
Smoking status, n (%):						
Never	160(93.0)	181(91.4)		56(57.7)	52(46.9)	
Past smoker	11(6.4)	16(8.1)	0.821	21(21.7)	30(27.0)	0.293
Current smoker	1(0.6)	1(0.5)		20(20.6)	29(26.1)	
Fruit (times/week)	3(2-6)	2(1-5)	0.078	2(1-6)	2(1-3)	0.016
Vegetables (times/week)	6(3-9)	4(2-6)	<0.0001	4(2-6)	3(2-6)	0.189
PAEE (KJ/Kg/day)	54.3±21.1	38.4±16.6	<0.0001	65.7±26.2	52.1±22.5	0.0001
Sedentary time (min/day)	925.1±143.7	1016.4±137.3	<0.0001	880.9± 154.1	960.9±149.4	0.0005
LPA time (min/day)	379.01±95.7	333.1±104.1	0.0001	414.3±98.7	366.4±111.7	0.003
MVPA time (min/day)	116.2(69.6-201.6)	75.3(47.5-113.2)	<0.0001	132.7(61.8-202.1)	94.2(54.7-148.3)	0.038
GPAQ PAEE (KJ/Kg/day)	82.5(7.2-178.6)	7.6(3.4-53.8)	<0.0001	38.5(3.2-141.1)	18.8(3.5-74.4)	0.177
GPAQ work (MET-min/week)	7480(0-16800)	0(0-4800)	<0.0001	2880(0-12960)	0(0-6000)	0.027

GPAQ leisure (MET-min/week)	0(0-0)	0(0-0)		0(0-0)	0(0-0)	
GPAQ travel (MET-min/week)	1680(560-3360)	720(300-1680)	<0.0001	840(420-5040)	840(280-2520)	0.276

Results are presented as arithmetic mean [or median (25th-75th percentile) for non-normally distributed variables] or n (%). p-values are from a t-test for normally distributed continuous variables (or Mann Whitney test for non-normally distributed variables) and from a chi-squared test for categorical variables.

PAEE, physical activity energy expenditure; LPA, light physical activity; MVPA, moderate to vigorous physical activity; GPAQ, global physical activity questionnaire

Table S2: Metabolic characteristics of the study population stratified by sex and urban/rural residence, Cameroon study (n=578)

Characteristics	Women (n=370)			Men (n=208)		
	Rural (n=172)	Urban (n=198)	p-value	Rural (n=97)	Urban (n=111)	p-value
Serum folate (nmol/L)	14.4(9.29-24.3)	11.4(8.31-16.1)	0.0006	18.1(11.3-27.1)	10.7(7.09-15.3)	<0.0001
Serum folate including meFox (nmol/L)	18.0(12.8-30.1)	14.9(10.8-21.0)	0.0002	25.1(14.5-35.1)	13.6(8.89-20.3)	<0.0001
meFox (nmol/L)	3.96(2.79-5.77)	2.96(1.95-4.98)	0.0001	5.54(3.18-8.97)	2.84(1.98-4.85)	<0.0001
Folate deficiency, n (%)	51(29.7)	82(41.4)	0.019	19(19.6)	52(46.9)	<0.001
HoloTC (pmol/L)	69.7±32.1	80.4±34.6	0.0028	69.9±34.5	78.7±35.9	0.085
HoloTC deficiency (n=547), n (%)	49 (29)	35(18.9)	0.026	33(36.3)	28(27.5)	0.189
BMI (kg/m^2)	24.9±4.9	29.1±5.5	<0.0001	23.1±3.3	25.4±4.1	<0.0001
BMI categories, n (%)<br><25<br>25-29.9<br>≥ 30	 103(59.9)<br>41(23.8)<br>28(16.3)	 50(25.3)<br>56(28.3)<br>92(46.5)	<0.001	 72(54.2)<br>22(22.7)<br>03(3.1)	 65(58.6)<br>35(31.5)<br>11(9.9)	0.03
Waist circumference (cm)	85.3±11.4	94.1±12.9	<0.0001	82.9±8.0	89.2±11.3	<0.0001
Central obesity, n (%)	114 (66.3)	167(84.3)	<0.0001	9(9.3)	30(27.0)	0.001
Waist to hip ratio	0.84±0.08	0.85±0.07	0.89	0.86±0.05	0.87±0.06	0.094
Body fat (%)	30.3±8.3	37.4±7.59	<0.0001	15.9±5.5	20.1±7.0	<0.0001
Systolic blood pressure (mmHg)	117 ± 19	124 ± 22	0.003	121 ± 15	130 ± 23	0.0007
Diastolic blood pressure (mmHg)	74±12	80 ± 14	0.0001	72 ± 12	78 ± 15	0.003
Fasting blood glucose (mmol/L)	4.82±1.39	4.86±1.08	0.752	4.68±1.49	4.65±1.56	0.89
2-h blood glucose (mmol/L)	6.23±1.74	6.61±1.93	0.054	5.91±1.55	6.07±1.99	0.54
Total cholesterol (mmol/L)	3.85±0.95	4.02±0.99	0.099	3.64±0.97	3.74±0.95	0.442

LDL cholesterol (mmol/L)	2.26±0.83	2.36±0.85	0.244	2.08±0.80	2.13±0.83	0.664
HDL cholesterol (mmol/L)	1.19±0.33	1.26±0.32	0.027	1.19±0.36	1.23±0.31	0.437
Triglycerides (mmol/L)	0.78(0.63-1.01)	0.71(0.57-0.91)	0.03	0.71(0.58-0.93)	0.73(0.55-0.97)	0.933
Fasting insulin (pmol/L)	22.3(14.2-39.1)	24.5(13.8-37.7)	0.799	14.8(6.5-27.4)	17.3(8.1-28.9)	0.391
CRP (mg/L)	5.26(2.61-8.41)	4.6(2.64-8.33)	0.699	5.56(2.58-9.89)	4.22(2.12-8.15)	0.099
Adiponectin (µg/ml)	6.85(4.63-9.34)	5.88(3.72-7.96)	0.0006	4.34(3.09-6.34)	4.40(3.07-5.87)	0.946
HOMA-IR index	0.77(0.47-1.47)	0.87(0.49-1.32)	0.706	0.49(0.20-0.86)	0.60(0.23-1.08)	0.365
Metabolic syndrome score	-0.31±2.25	0.35±2.67	0.013	-0.58±2.32	0.38±2.83	0.009

Results are presented as arithmetic mean [or median (25th-75th percentile) for non-normally distributed variables] or n (%). p-values are from a t-test for normally distributed continuous variables (or Mann Whitney test for non-normally distributed variables) and from a chi-squared test for categorical variables.

meFox, oxidation product of 5-methyltetrahydrofolate (pyrazino-s-triazine derivative); physical activity energy expenditure; HoloTC, holotranscobalamin; BMI, body mass index; LDL, low density lipoproteins; HDL, high density lipoproteins; CRP, C-reactive protein; HOMA-IR, homeostatic model assessment of insulin resistance

Table S3: Characteristics of participants with vitamin B12 and folate deficiency, Cameroon study (n=547)

Characteristics	Combined deficiency, n=37 (6.8%)	Others, n=510 (93.2%)	p-value
Age (years)	36.3± 9.1	38.4 ± 8.5	0.15
Sex, n(%)			
Male	16(43.2)	177(34.7)	0.29
Education level, n(%)			
< primary education	5(13.5)	95(18.7)	
Primary	16(43.2)	225(44.2)	0.814
Secondary and high school	10(27.1)	125(24.5)	
University	6(16.2)	64(12.6)	
Site, n(%)			
Urban	27(73.0)	260(51.0)	0.01
Alcohol consumption (%):			
Never	3(8.1)	57(11.2)	
Past	4(10.8)	50(9.8)	0.850
Current	30(81.1)	403(79.0)	
Smoking status, n(%):			
Never	26(70.3)	396(77.7)	
Past	7(18.9)	69(13.5)	0.572
Current	4(10.8)	45(8.8)	
PAEE (KJ/Kg/day)	50 ± 18.1	50.9 ± 23.4	0.417
GPAQ PAEE (KJ/Kg/day)	6.5(3.5-75.3)	25.5(4.2-128.9)	0.166
Fruits (times/week)	3(1-6)	2(1-5)	0.539
Vegetables (times/week)	4(2-8)	4(2-7)	0.914
BMI (Kg/m^2)	26.1 ± 4.8	26.1 ± 5.3	0.97
Waist (cm)	89.6 ± 11.3	88.5 ± 12.2	0.617
Systolic blood pressure (mmHg)	123 ± 25	122 ± 20	0.786

Diastolic blood pressure (mmHg)	78 ± 16	76 ± 13	0.529
Fasting blood glucose (mmol/L)	4.9 ± 1.3	4.8 ± 1.4	0.67
2-h blood glucose (mmol/L)	6.15 ± 1.46	6.28 ± 1.87	0.682
HOMA-IR	0.45(0.87-1.41)	0.71(0.38-1.23)	0.27
Metabolic syndrome z-score	0.18 ± 2.7	-0.007 ± 2.5	0.672

Results are presented as arithmetic mean [or median (25th-75th percentile) for non-normally distributed variables] or n (%). p-values are from a t-test for normally distributed continuous variables (or Mann Whitney test for non-normally distributed variables) and from a chi-squared test for categorical variables.

Vitamin B12 deficiency was defined as holotranscobalamin less than 50 pmol/L and folate deficiency as serum folate less than 10 nmol/L

PAEE, physical activity energy expenditure; GPAQ, global physical activity questionnaire, BMI, body mass index, HOMA-IR, homeostatic model assessment of insulin resistance

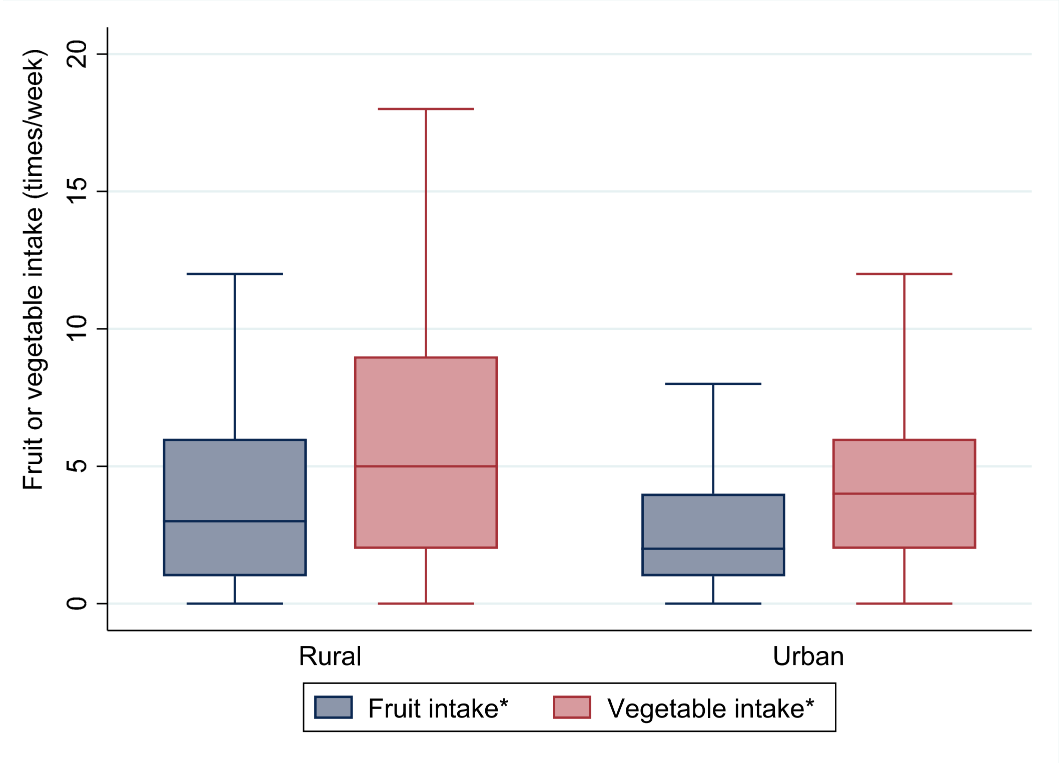

Figure S1: Distribution of self-reported fruit and vegetables intake in rural and urban residents (Cameroon study, n=578)

Median (interquartile range)

*P<0.05 for difference in self-reported intake between participants living in rural and urban areas using a Mann Whitney test

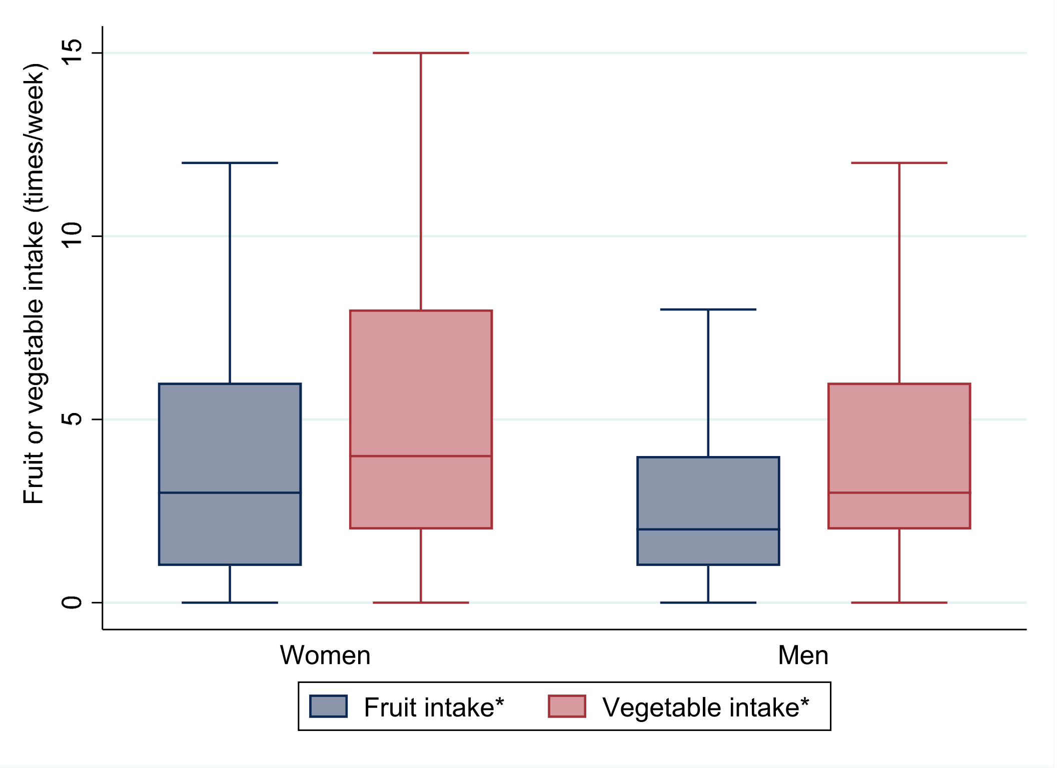

Figure S2: Distribution of self-reported fruit and vegetables intake in women and men (Cameroon study, n=578)

Median (interquartile range)

*P<0.05 for difference in self-reported intake between women and men using a Mann Whitney test

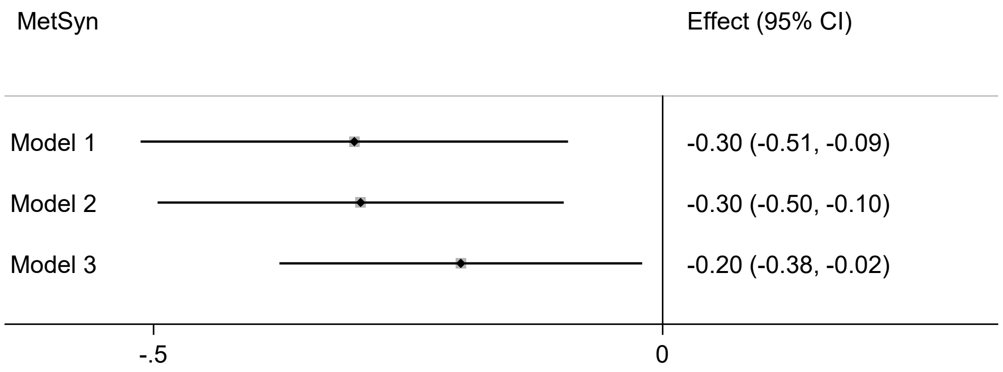

Figure S3: Association between serum folate and metabolic syndrome score (Cameroon study, n=520).

MetSyn, metabolic syndrome score; effect (β-coefficient), difference in metabolic syndrome score per 1SD (10.8 nmol/L) of serum folate; 95% CI: 95% confidence interval

Model 1: Unadjusted

Model 2: Adjusted for age, sex, level of education, smoking, alcohol intake, residential site

Model 3: model 2 + body mass index + objectively measured physical activity

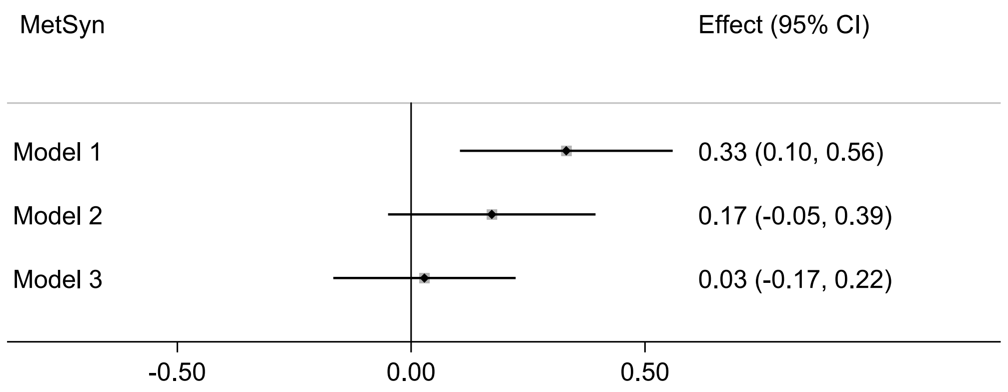

Figure S4: Association between serum holotranscobalamin and metabolic syndrome score (Cameroon study, n=491)

MetSyn, metabolic syndrome score; effect (β-coefficient), difference in metabolic syndrome score per 1SD (34.3 pmol/L) of serum holotranscobalamin; 95% CI: 95% confidence interval

Model 1: Unadjusted

Model 2: Adjusted for age, sex, level of education, smoking, alcohol intake, residential site

Model 3: model 2 + body mass index + objectively measured physical activity
